# Consecutive Spawnings of Chinese Amphioxus, *Branchiostoma belcheri*, in Captivity

**DOI:** 10.1371/journal.pone.0050838

**Published:** 2012-12-12

**Authors:** Guang Li, Xi Yang, ZongHuang Shu, XiaoYing Chen, YiQuan Wang

**Affiliations:** 1 School of Life Sciences, Xiamen University, Xiamen, People's Republic of China; 2 Shenzhen Research Institute of Xiamen University, Shenzhen, People's Republic of China; Sars International Centre for Marine Molecular Biology, Norway

## Abstract

Cephalochordate amphioxus is a promising model animal for studying the evolutionary and developmental mechanisms of vertebrates because its unique phylogenetic position, simple body plan and sequenced genome. However, one major drawback for using amphioxus as a model organism is the restricted supply of living embryos since they are available only during spawning season that varies from a couple of days to several months according to species. Therefore we are aiming to develop methods for obtaining viable amphioxus embryos in non-spawning season. In the current study, we found that *Branchiostoma belcheri* could develop their gonads and spawn consecutively in the laboratory when cultured in a low density at a high temperature (25–28°C) supplied with sufficient food and proper cleanness. Among the approximate 150 observed animals, which spawned spontaneously between November and December 2011, 10% have spawned twice, 10% three times, and 80% four times, through April 2012. The quality and quantity of the gametes reproduced in the consecutive spawning have no obvious difference with those spawned once naturally. Spawning intervals varied dramatically both among different animals (from 1 to 5 months) and between intervals of a single individual (from 27 to 74 days for one animal). In summary, we developed a method with which, for the first time, consecutive spawnings of amphioxus in captivity can be achieved. This has practical implications for the cultivation of other amphioxus species, and eventually will greatly promote the utilization of amphioxus as a model system.

## Introduction

Amphioxus (also called lancelet) belongs to the subphylum Cephalochordata which, together with the subphylum Urochordata (tunicates), represent the closest living invertebrate relatives of the vertebrate. Compared to the much derived tunicates, amphioxus features remain more vertebrate-like in either morphology or genomic sequence. Like vertebrates, amphioxus possesses a hollow nerve cord, a dorsal notochord, segmented muscles and pharyngeal gill slits, but lacks several vertebrate-specific characteristics including paired sensory organs or a cartilaginous or bony skeleton [1]. The amphioxus genome has not undergone two-round whole genome duplications which occurred early in the evolution of vertebrates [2], [3], and retains more archetypal features of ancestral chordates in comparison with either vertebrates or tunicates [2], [4]–[6]. Given these advantages, amphioxus has long been considered as an ideal model animal for studying vertebrate evolution and development [7]–[9]. Several important findings including resolution of the debate on two-round whole genome duplication [2], and the probable mechanism from which the vertebrate neural crest was generated [10] have been achieved by studying amphioxus. Moreover, the newly refined phylogeny of chordates, which places cephalochordates as the basal lineage of the chordate phylum [2], [11], makes amphioxus not only a model for studying the evolution of vertebrates, but also for better understanding the elaboration of the entire chordate lineage.

To date, a total of about 30 amphioxus species have been recorded around the world [12], [13]. Four of them have been commonly used for biological studies (two congeners *Branchiostoma belcheri* and *B. japonicum* were wrongly treated as the same species previously [14], [15]). They are the Floridian-Caribbean *B. floridae*, the European *B. lanceolatum*, and the East Asian *B. belcheri* and *B. japonicum*. Because of great interests among the scientific community, several milestones have been achieved recently in amphioxus researches, which include the completion of the genome sequencing projects of *B. floridae* and *B. belcheri*, the development of microinjection technique in *B. floridae* [9], [16], *B. belcheri* and *B. japonicum* (our unpublished data), and the establishment of methods for rearing *B. belcheri* and *B. japonicum* in capacity [17], [18] and *B. lanceolatum* in artificial sea water [19], [20], and method for spawning inductions of *B. lanceolatum* [21], [22], *B. belcheri* and *B. japonicum* (our unpublished data).

All these achievements have greatly promoted amphioxus to be used as a model system. However, lack of living embryos in non-spawning season is still one of the major impediments for the utilization of amphioxus in developmental studies, since those materials are available only during the spawning season, which varies from a few contiguous days for *B. japonicum* [23] to approximately one month for *B. lanceolaten* [21]. This drawback has also affected the development of experimental techniques in these animals, which again hinders the development of amphioxus as a model system. For these reasons, studies on amphioxus are restricted mainly to expression patterns either by *in situ* hybridization or by immunohistochemical staining, although gene functional studies have been carried out occasionally through activating or inhibiting specific signaling pathways using small chemical molecules and synthesized proteins [9]. To solve the problem, we report the establishment of a method to spawn *B. belcheri* consecutively in the laboratory.

## Materials and Methods

### Animal culture in the laboratory

Adult *B. belcheri* animals were cultured using a procedure described previously (Zhang et al., 2007) with several modifications as follow. First, the water temperature was maintained between 25 to 28°C by controlling the room temperature and salinity were kept between 2.5 to 3.0% by adding fresh water or sea salt. Second, the sand used is very fine, being sifted through 1 mm wire sieve; and the sand layer for animal settlement is about 2 centimeters thick. Third, the photoperiod is controlled with 13.5-hr light (23:00–13:30) and 10.5-hr dark (13:30–23:00). Fourth, the sand and containers were cleaned once a week to keep a clean living environment as described below. Before the old water was poured out, the container inner wall was wiped with a cloth towel to remove the algae adhering to the wall, and the sand was disturbed gently by hand to wash out the debris buried in the sand. Then the dirty water was poured out immediately into a barrel covered with a filter (to collect the unsettled animals). The sand and containers were cleaned at least twice using freshly-filtered seawater, and the animals in the filter was transferred back into the container and fresh seawater was added until the container is three-fourths full. Last, the animals were fed three times a day with a mixture of *Isochrysis galbana*, *Dicrateria zhangjiangensis* and *Chaetoceros muelleri* (unless otherwise specified) at 9:00–10:00, 14:30–15:00 and 20:00–20:30, respectively. The final concentration of the mixed algae is about 10^4^–10^5^ cells/ml, and the ratio of different algae varies according to season.

#### Experiment I

In our previous observation, we noticed that some individuals of *B. belcheri*, both in the field and in the laboratory, could spawn twice in one spawning season with an interval of about one month. This observation led us to hypothesize that, if reared in appropriate environmental conditions, amphioxus could probably spawn multiple times within and out of the breeding season. To test this hypothesis, two groups of animals, which spawned on October 30th (Group I, including 24 females and 24 males) and November 14th (Group II, including 22 females and 43 males) of 2011, were collected and reared in 5 litre red plastic barrels (their diameters and depths are about 20 and 18 centimeters, respectively) supplied with continuous bubbling air. No more than 25 individuals were kept in each barrel, and the seawater was about 3–4 litres. These animals were named “First Spawner” animals, and their gonadal recrudescence was examined visually every ten to fifteen days. After each examination, the animals with medium or large redeveloped gonads were collected, counted and reared in new 5 litre red plastic barrels (no more than 25 individuals per barrel). These animals with redeveloped gonads were called “Second Development” animals, and their spawning was also examined visually every ten to fifteen days, supplemented with a daily check in the afternoon. On the spawning and checking days, animals were screened out from the sand, and the ones with small or empty gonads were collected, counted and raised in another set of 5 litre red plastic barrels. Those individuals were roughly regarded as spawned on the day when we made the observation. In order, these secondly-spawned animals were named “Second Spawner” animals. Consequently, we have “Second Development”, “Third Spawner”, “Third Development” animals, and so on.

#### Experiment II

This experiment was conducted to track the consecutive spawning behavior of *B. belcheri* individually. Sixteen males, nineteen females and two hermaphrodites, which were induced to spawn by heat-shock on dates between Nov. 23^rd^ and Dec. 24^th^, 2011, were collected and reared individually in 500 ml plastic beakers (their diameters and depths are about nine and twelve centimeters respectively) without bubbling air. The seawater in the barrel was kept about 300 ml (the animal would jump out if the water is too much). To record their gonad development processes and spawning behaviors, the first four to six gonads on the right side of the bodies of these animals were photographed every ten to fifteen days under a stereomicroscope. Gonads becoming obviously emptied or smaller than last recorded were adopted as the marker of spawning, and the spawning date was roughly regarded as the same day of the observation. The spawnings were also estimated by checking whether the water in the beakers contained eggs (for females) or became cloudy (for males) before the daily late afternoon feeding. We also made additional short-time span records (3 to 4 days at the beginning and 6 to 9 days later) to see more details on the gonadal development of 15 individuals that released their gonads on 15th May 2012.

#### Experiment III

The gonads of a ripe amphioxus weigh nearly half of the whole animal body weight. Thus, amphioxus needs to accumulate lots of nutrition from food to develop mature gonads. High-protein food made from animal debris (like fish) has been shown to accelerate the growth and gonad maturation in many commercial shrimps or fishes aquacultures. Therefore, we carried out the following experiment to examine whether food made from animal debris could accelerate the development of amphioxus gonads. For this purpose, a total of 104 adult *B. belcheri* were collected from Huangcuo, Xiamen (Fujian, China) on November 28^th^ 2011, which is about three months after the breeding season of this species in the field, thus no visible gonads were observed in them. The 104 animals were randomly divided into two groups: one group of 48 individuals was fed with mixed fresh algae plus about 1 gram commercial shrimp flakes (Sailboat Brand, produced by Bonasse Biochemistry Technology Enterprise Co., Ltd, Taipei), and another group of 56 individuals was fed with mixed fresh algae only. The chips were washed out through 300 mesh nylon net with the aglae mixture solution before being fed to the animals. Other rearing conditions (such as temperature, density, *etc.*) and the data collection were the same as those described in Experiment I. This experiment could also tell us how many days are needed for animals collected from the field in non-breeding season (without obvious gonads) to develop their gonads and spawn under the conditions adopted in Experiment I.

### Quantity evaluation of eggs and embryo surviving rates in multiple reproductions

The numbers of eggs produced by several animals from Experiment II were counted to evaluate the egg quantity. On a couple of spawning days, blastulas (about 200 for each time) from Experiment I and III were collected and raised in six-well dishes at 25°C without light in an incubator. Their surviving rates at the following developmental stages were recorded: gastrula, hatching neurula, mouth-opened larvae and larvae of 3-gill slits.

### Histological section and staining

Animals were cut into small segments (about 1 centimeter each) and fixed in 4% PFA (paraformaldehyde) in PBS (phosphate buffer, pH = 7.4) at 4°C overnight. After dehydration in an ascending series of ethanol and clearing in xylene, the tissues were embedded in paraffin, sectioned transversally at 5 µm, and mounted on glass slides (RNase-free) pre-coated with poly-L-lysine. Section staining was performed according to conventional HE-staining protocols.

## Results

### Consecutive spawnings of *B. belcheri* in captivity

**Figure 1:**
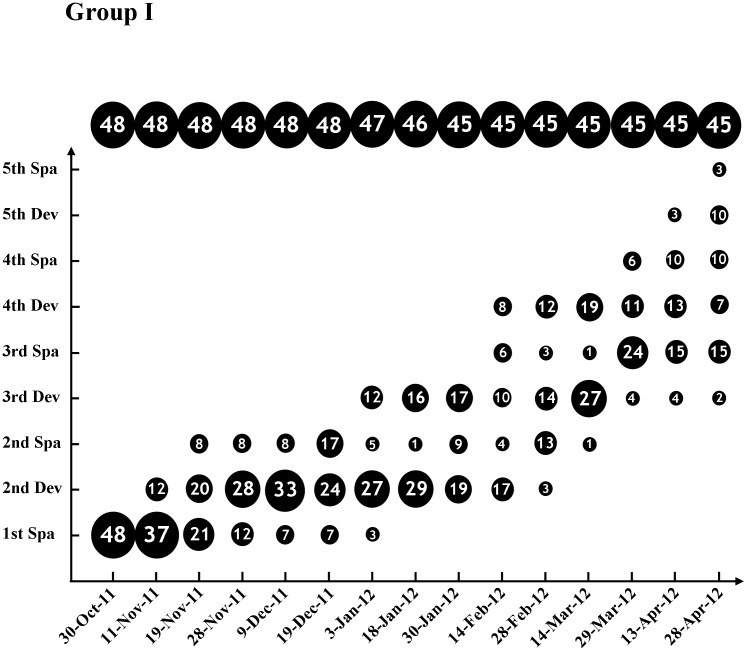
The consecutive gonadal development and spawning of *B. belcheri* in the Group I of Experiment I. Animal numbers of different gonadal phases on each data collection day are shown on the diagram roughly on scale. The figures at the top of the diagram are the total animal number on each data collection day. Abbreviations: Dev, Development; Spa, Spawning.

**Figure 2:**
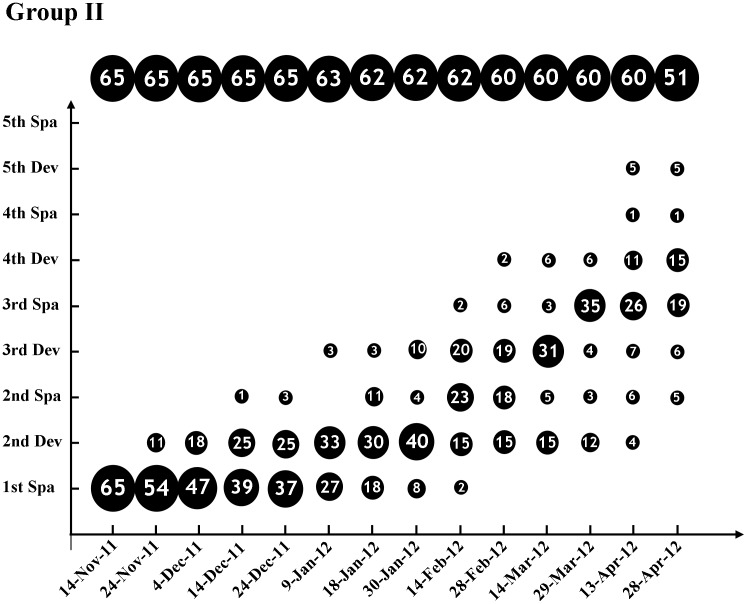
The consecutive gonadal development and spawning of *B. belcheri* in the Group II of Experiment I. Animal number of different gonadal phases on each data collection day are shown on the diagram roughly on scale. The figures at the top of the diagram are the total animal number on each data collection day. Abbreviations: Dev, Development; Spa, Spawning.

Our previous study, on a population level, suggested that a portion of adult *B. belcheri* could spawn multiple times in a given breeding season [24]. This finding indicated that *B. belcheri* might spawn consecutively within and out of the breeding season if reared under appropriate conditions. We tested this hypothesis in Experiment I. As shown in Fig. 1, all of the 48 examined animals in Group I had gonad recrudescence following the first spawning on 30^th^ of October, 2011. In addition, most animals (43/45 = 95.6%) spawned at least three times by April 28^th^ 2012 (181 days later) when the last data was collected. Among them, 26.7% (12/45) individuals entered into developmental stages of the Fifth Development or Fifth Spawning (see the definitions in Material and Methods section). In Group II (Fig. 2), except for two animals whose gonads did not redevelop again even after three-month cultivation and thus were excluded from our subsequent analysis, all other 60 individuals refilled their gonads, with most of them (43/60 = 71.7%) being in or after the Third Spawning stage before April 13^th^ 2011. These results not only proved that nearly all adult *B. belcheri* could spawn consecutively within or out of the breeding season if cultured under proper environment conditions (see Materials and Methods for details), but they also showed the fact that the intervals between two successive spawnings diverged very much among individuals (see Table 1). The mean intervals were between 80 and 104 days for Group I and II animals, respectively (Table 1). No obvious difference was observed in the speed of gonadal recrudescence between the males and females in Group I, but the males in Group II appeared to develop faster than females (Table 2). This inconsistency might be caused by either reproductive diversity among different individuals or the biased sex ratio in Group II (41 males vs 20 females).

**Table 1:** Interval span (days) between consecutive spawnings in Experiment I.

Group	Duaration (days)	No. of spawnings	Mean interval (days)	No. of animals	mean interval (days)
I	181	1	181.0	2	80
	181	2	90.5	22	
	181	3	60.3	19	
	181	4	45.3	2	
II	166	0	166.0	4	104
	166	1	166.0	13	
	166	2	83.0	37	
	166	3	55.3	6	

Note: the first spawning is not counted.

**Table 2:** Sex ratio of animals in Experiment I after six-month culture.

Group	Sex	Total number	2nd Dev		2nd Spa		3nd Dev		3nd Spa		4th Dev		4th Spa		5th Dev	
			Number	Percentage	Number	Percentage	Number	Percentage	Number	Percentage	Number	Percentage	Number	Percentage	Number	Percentage
I	Male	21	-	-	-	-	2	9.52%	8	38.10%	5	23.81%	5	23.81%	1	4.76%
	Female	24		-	-	-	2	8.33%	7	29.17%	8	33.33%	5	20.83%	2	8.33%
II	Male	40	1	2.50%	5	12.50%	6	14.00%	13	32.5%	10	25.00%	1	2.50%	5	12.50%
	Female	20	3	15.00%	2	10.00%	1	5.00%	13	65.00%	1	5.00%	-	-	-	-

### Consecutive spawning behaviors of *B. belcheri* individuals

**Figure 3:**
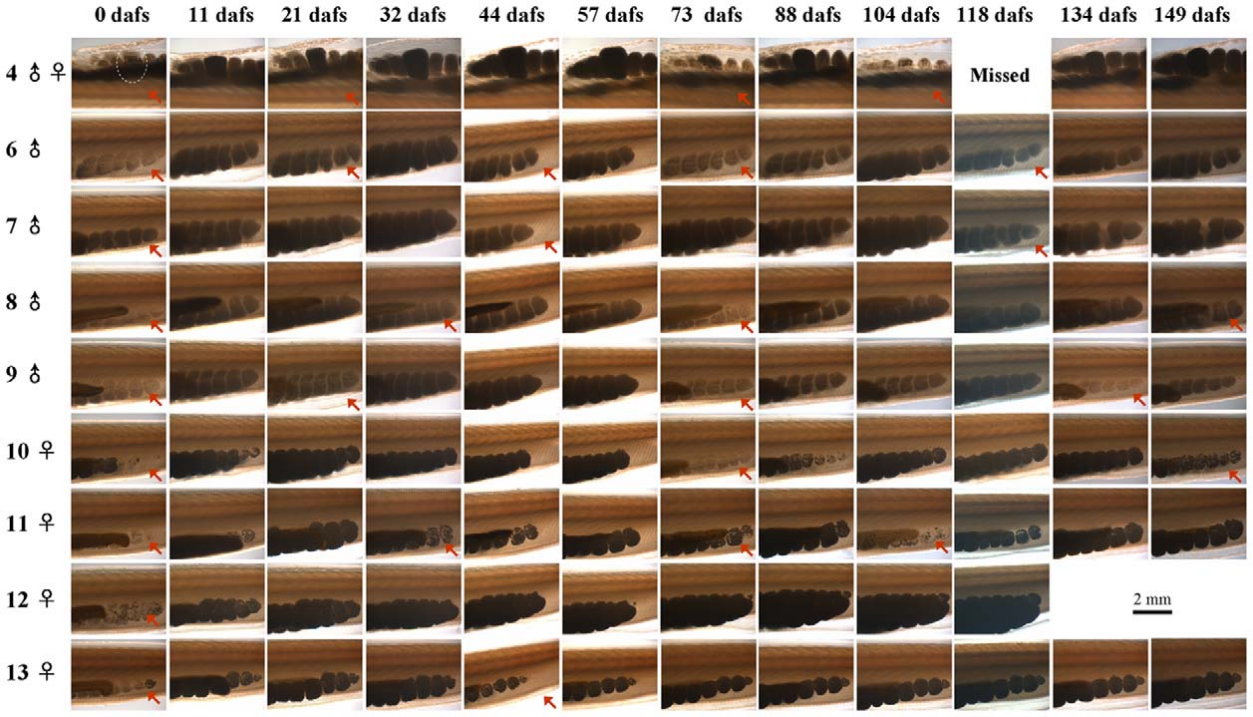
The consecutive gonadal development and spawning of individual *B. belcheri* in Experiment II. The animal anterior is shown to the right, and the belly faces down (except for hermaphrodite #4). The ovary of hermaphrodite #4 is highlighted by a white dotted circle. Red arrows indicate that the spawning occurred. The days of photographing these animals were shown at the top of the figure and the first spawning day was defined as zero dafs (day after first spawning). It should be noted that hermaphrodite #4 spawned ten days earlier than the other eight animals for their first spawning, and the information shown at the top of the figure is derived from the latter, thus the numbers should be plus 10 for hermaphrodite #4. Female #12 died after 118 dafs.

**Figure 4:**
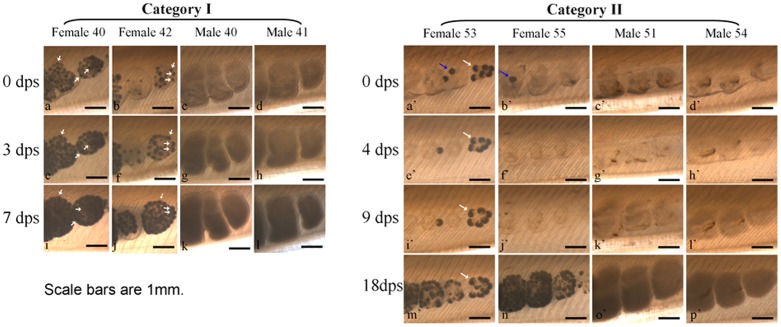
The fine records of *B. belcheri* gonadal redevelopment. Animals from Category I are shown on the left and animals from Category II are shown on the right. These animals were collected from our large scaled cultures (under conditions similar to those applied in the study) on 15th of May 2012, and they spawned at least two times after November 2011. White arrows indicate the preserved large oocytes in the following gonadal recrudescence, and blue arrows mark the ongoing reabsorption bed of large oocytes. Abbreviation: dps, day post spawning.

**Figure 5:**
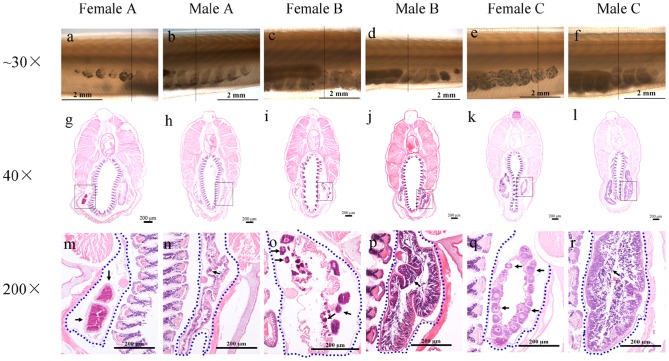
The newly spawned amphioxus *B. belcheri* and their sections. Animal A is a typical Category II animal, animal B is a typical Category I animal, and animal C is Category I animal one day post spawning. Vertical lines in the photos at the top line (a–f) indicate where the sections in the middle line (g–l) were performed and the figures (m–r) shown at the bottom are higher magnifications of the regions highlighted by black boxes on the sections in the middle. The gonads in the figures at the bottom are highlighted with blue dotted lines. Arrows in m indicate the unspawned ripe eggs of Female A; arrow in n indicates the thin germinal epithelium (with few spermatocytes inside) of Male A; arrows in o indicate the unreleased immature small oocytes (5–50 micrometer in diameter) of Female B; arrow in p shows many unspawned spermatocytes of Male B; arrows in q indicate the redeveloping oocytes of Female C; and arrow in r indicates redeveloping spermatocytes of Male C.

We carried out another experiment (Experiment II) to further analyze the consecutive spawning behavior of *B. blecheri*. Animals spawned between November 23^rd^ and December 26^th^ 2011, and on May 15^th^ 2012, were reared individually in 500 ml beakers, under similar conditions as Experiment I but without an air supply. By doing this, we could follow and record the gonad developmental process and consecutive spawning behavior of each individual. Several important findings were observed in this experiment. First, we found that all examined animals (as was the case for animals in the other two experiments) expelled most of their gametes in each spawning (Fig. 3, red arrows, Fig. 4a–d and a'–d' and Fig. 5a–d). In some individuals, the remaining gonads (Category I) appeared as large and honeycomb-like (female) or cloudy (male) gonad debris and obviously contains many small oocytes or spermatocytes respectively (Fig. 4 a–d and Fig. 5c–d); while in the others (Category II), the gonads appeared as small and transparent gonad remnants (Fig. 4a'–d' and Fig. 5a–b). Sections of these newly spawned animals further depicted the fine structures of nearly emptied gonads (Fig. 5). Females of Category I have many relatively small oocytes (5–50 micrometer in diameter) in their gonad remains (Fig. 5o, black arrow), but for females of Category II only unreleased ripe eggs were observed (Fig. 5m, black arrow). Similarly, males of Category I seemed to have a thick germinal epithelium surrounding the central lumen of different staged gametes (Fig. 5p, black arrow), while males of Category II had only a very thin germinal epithelium which obviously only had a few spermatocytes inside (Fig. 5 n, black arrow). Second, *B. belcheri* could redevelop their gonads and spawn consecutively when they were reared individually (Fig. 3 shows some of these cases). An even shorter spawning intervals (65–70 days in average) (Table 3) were observed compared to those obtained in Experiment I (80–104 days in average) (Table 1). Interestingly, it appears that animals belonging to Category I could redevelop their gonads directly at the basis of their gonad remains (Fig. 4 a–l), but the ones in Category II could restart gonadal development only after partially reabsorbing their gonad debris (Fig.4 a'–p'). We also found that at the early stage of gonadal redevelopment the unreleased large oocytes (over 100 micrometer in diameter) were completely preserved and redeveloped along with the small oocytes in Category I animals (Fig.4a, c, and i and b, f and j, white arrows), but some of them were reabsorbed or released in Category II animals (Fig. 4a'–b', blue arrows). However, if the animal was reared at a low temperature (19°C) or without food supply, all these animals would reabsorb their gonad remnants slowly (about one month) and not redevelop their gonads again (data not shown). Third, the intervals of consecutive spawning frequency varied greatly among different individuals (Table 3) as we observed in Experiment I. The shortest interval observed was 12 days, which happened between the first and second spawning in female #14. The longest interval was from female #7: by April 30^th^ 2012 (158 days after its first spawning), it had not spawned again although its gonads had grown very big. This variation was also observed in the same individual (Table 3). For instance, the intervals from the first to second and third to fourth spawning of male #8 were 25 and 76 days respectively. In general, animals spawned 3 times more (e.g. male #6 and #9, female #2, #11, #15 and #19, and hermaphrodite #4) always had shorter intervals, range from 25 to 60 days. Fourthly, for the first time we made a close observation of the spawning behavior of hermaphroditical amphioxus. The two examined hermaphrodites, like most reported previous [25], [26], both had more ovaries than testis. Within the five months of the experiment, hermaphrodite #4 spawned three times, and each time the eggs and sperms were released simultaneously, as evidenced by the presence of fertilized eggs and the emptied ovaries and testis (Fig. 3, pictures on the first line). In contrast, hermaphrodite #10 just spawned only one additional time (Table 3), and only the sperms were partially released (data not shown).

**Table 3:** Mean span (days) between consecutive spawnings of animals in Experiment II.

Individuals	No. of Spawnings	Interval (days) between spawnings					Individuals	No. of Spawnings	Interval (days) between spawnings			
		1th to 2nd	2nd to 3rd	3rd to 4th	4th to 5fh	Mean			1th to 2nd	2nd to 3rd	3rd to 4th	Mean
1♂^a^	0						1♀	2	44	80		62
2♂	2	54	60			57	2♀	3	21	23	44	29
3♂	2	42	92			67	3♀	1	128			128
4♂♀	3	31	52	31		38	4♀^b^	1	35			35
5♂^b^	1	35				35	5♀	2	128	31		80
6♂	4	21	23	29	45	30	6♀	1	113			113
7♂	3	44	44	30		39	7♀	0				158
8♂	3	25	48	76		50	8♀	1	53			53
9♂	3	21	52	31	30	34	9♀	2	51	60		56
10♂♀	1	124				124	10♀	2	73	76		75
11♂	1	121				121	11♀	3	32	41	31	35
12♂^a^	0						12♀^a^	0				
13♂	0	136				136	13♀	1	44			44
14♂	2	56	45			51	14♀^c^	2	12	32		22
15♂	1	56	61			59	15♀	3	31	60	45	45
16♂	1	98				98	16♀^c^	2	53	31		42
17♂	2	36	61			49	17♀^a^	0				
18♂	0	129				129	18♀	1	98			98
							19♀^d^	3	23	29	45	32
all						70						65

Note:

a animals died before the second spawning;

b, c and d animals died after the second, third and fourth spawning respectively. The first spawning is not counted.

### The breeding season of animals collected from the field could be advanced by this method

To examine the effect of high-protein commercial food on amphioxus gonad development and to see whether our method could advance the onset of the breeding season for wild animals, we performed Experiment III. Our results shown that, early gonad development was slightly slower in animals fed with mixed fresh algae plus a certain quantity of shrimp chips than those fed with mixed fresh algae only. However, the late gonad development appeared quicker and spawned earlier in the animals fed with the food supplemented with shrimp chips. For example, 89.3% (50/56) and 100% (56/56) of animals fed with mixed fresh algae had obvious refilled gonads, and the corresponding numbers were 67.4% (31/46) and 80.4% (37/64) for animals fed with mixed fresh algae plus shrimp flakes by 14th and 30th of January, 2012. However, 58.8% (30/51) of animals in the former group had spawned, and 81.5% (35/41) spawned in the latter group by April 28^th^ 2012. These results also demonstrated that wild animals (without gonads) could become mature again in non-breeding season when they were reared in appropriate conditions in the lab. All animals in this experiment were mature and 69.9% (65/93) of them had spawned by May 6^th^ 2012. By contrast, the gonads of all captured wild *B. belcheri* in the field at the time were not discernible with the naked eye (or at very early developmental stages), which was consistent with our previous observations [15].

### Numbers of eggs and survival rates of embryos in consecutive spawning

The numbers of egg and subsequent survival rates of embryos in consecutive spawnings are important for developmental studies. Numbers of eggs released each time were around 3, 3.5 and 11 thousands, respectively in three randomly selected spawnings (second, third and second spawning of female #1, 2 and 3 from Experiment II), which were equivalent to that in our previous observation [17]. We also observed that most animals, which spawned more than once from Experiment II, appeared to have similar gonad size before each spawning (Fig. 3). This result indicated that the numbers of egg produced in consecutive spawning should be similar to that in the initial or natural spawning. As shown in Table 4, the embryo survival rates at stages of hatching neurula, mouth-opened and 3-gill slit larvae were 63.8%–94.1%, 85.6%–47.1% and 79.1%–42.1%, respectively, which was consistent with those in a previous report [17]. The lower survival rates in the 5th spawning of animals in Group I of Experiment I (Table 4) are probably caused by too many sperms added for fertilization, since we found the water in the tank was cloudy before the embryos collection. Taken together, our results demonstrated that the quality and amounts of the gametes produced in the consecutive spawning had no obvious difference with those spawned in the normal breeding season.

**Table 4:** Embryo surviving rates in consecutive spawnings.

Sample resources	hatching neurula	mouth-opened larvae	3-gill-slit larvae
the first spawning of animals fed with mix algaes	88.5%	85.6%	79.1%
the 4th spawning of animals in Group I of Experiment I	94.1%	75.1%	64.5%
the 5th spawning of animals in Group I of Experimetn I	63.8%	47.1%	42.1%

## Discussion

### Factors affecting the consecutive reproduction of *B. belcheri*

Several factors have been suggested to affect amphioxus reproduction. *B. belcheri* spawns during the summer in its natural habitat, which indicates that a warm temperature (24–30°C) is a prerequisite to the activation of gonadal development and maturation [15]. As shown in Experiment III, the unseasonably warm temperature could advance the onset of *B. belcheri* spawning about five mouths earlier (from June to next January). The higher temperature was also found to be indispensable for the consecutive spawnings of *B. belcheri*, since animals raised in a lower temperature (19°C) after spawning could not redevelop their gonads as those reared at 25–28°C, even if provided with sufficient food and reared in low density (data not shown). Adequate food supply is another key factor affecting *B. belcheri* spawning. Nearly all of the lancelets examined in this study could become mature and spawn again after their first and subsequent spawning; however, gonads in these animals would degenerate when the amphioxus reared under similar conditions but without adequate food (data not shown). Food requirements have been suggested to be critical for the gonad maturation in other amphioxus species during their breeding seasons [21]. Low stocking density is another crucial factor for the consecutive spawning of *B. belcheri*, which is supported by our results obtained in Experiment I and II. Experiment II had a low stocking density (about 78.5 square centimeters per animal) and a short average interval (65–70 days, shown in Table 3) between spawnings of animals, while Experiment I had a relatively high stocking density (about 15.7 square centimeters per animal at the beginning) and a long average interval (80–104 days, shown in Table 1). We also observed a short spawning interval in amphioxus reared in a low density than the animals raised in a high density in another large scale culture (data not shown). The recommended stocking density should be at least 15 square centimeters per animal when the layer of the sand for animal settlement is about 2 centimeters thick. Finally, a clean settlement substratum is also very important, especially when spawning or animal death occurred. Otherwise, the water would become putrid very quickly under the high temperature and animals would die, as what happened in Group II in Experiment I between 14^th^ and 16^th^ April, 2012 (Fig. 2). The photoperiod might be essential as well, but whether it is favorable to the consecutive gonadal development of *B. belcheir* still requires further investigation.

### Important findings on *B. belcheri* consecutive spawning

During non-breeding season, the gonads of majority lancelets in their natural habitat are not observable by naked eyes or under a dissecting microscope. Their gonads begin to enlarge and eventually become mature and spawn prior to spawning season. *B. lanceolatum* [21], *B. floridae* [27] and *B. japonicum* [28] release most but not all of their gametes during each spawning. Similarly, we found that *B. belcheri* also did not shed all their gametes in either the first or successive spawning (Fig. 3, red arrows, Fig. 4a–d and a'–d' and Fig. 5a–d). After spawning, the gonads generally begin to degenerate and finally become invisible again at the end of spawning season in most lancelets both in their natural environment and in the laboratory. Multiple spawnings have been observed or suggested for most lancelet species including *B. belcheri* [15], *B. lanceolatum* [21], *B. floridae* [27], *B. japonicum* [26] and *Asymmetron lucayanum* [29]. However, most of these observations were based on studies at population levels, and consecutive spawning examined at individual levels has not been documented. By making close records on 15 *B. belcheri* individuals, we found that the spawned animals can be roughly divided into two categories according to the size and the amount of remaining gametes in the gonad debris: Category I with relatively larger and more gamete-containing gonads (Fig. 4a–d) and Category II with relatively smaller and less gamete-containing gonads (Fig. 4a'–d'). Intriguingly, animals from Category I could redevelop their gonads directly after spawning (Fig. 4a–i), but animals from Category II appear to reabsorb some of their gonad remnants first before restarting the gonadal developmental process (Fig. 4a'–p'). In the Category I animals, many small oocytes or spermatocytes of different stages were typically observed (Fig. 5o and p). The gonad recrudescence appeared to begin soon after spawning (Fig. 5q and r) and thus accounting for the observation of their gonadal redevelopment (Fig. 4a–i). However, no detectable oocytes (except for the unreleased large eggs) and few spermatocytes were revealed, respectively, in the gonads of females and males from Category II animals (Fig. 5m and n). Interestingly, some of the unreleased large eggs are not reabsorbed during gonadal degeneration (Fig. 5a', e', i' and m', white arrows), and they probably become mature again in the next round of gonad development.

Gamete quantity and quality are crucial for developmental studies on living embryonic materials. In this study, we found no obvious difference in the egg numbers and embryo survival rates produced in consecutive spawnings compared to those spawned during the typical spawning season. Moreover, our results also show that *B. belcheri* raised individually could develop their gonads and spawn normally, and none of them was found to be sex-reversed. These findings provide direct evidence for that *B. belcheri* adults could be sexually separated in large scale cultures like zebrafish, avoiding the laborious work in sexual identification since their gonads are usually unsexable by naked eyes. In previous studies, *B. japonicum* was shown to be unable to spawn spontaneously if being reared separately according to sex, although they could develop gonads normally [30]; but sexally-matured individual *B. lanceolatum* could spawn efficiently 36 hours following the separation [22]. Therefore the reproductive pattern described above appears to be slightly different among species.

### Variation of the consecutive spawning among different *B. belcheri* individuals

All lancelet species are wild, and thus the variation between individuals is very high. This was first demonstrated at genomic level: the allelic polymorphism rate is 3.7% at nucleotide level in *B. floridae* [2], and late at the growth rate of juveniles generated in the laboratory [17]. In this study, we found considerable varied spawning intervals among adult *B. belcheri* individuals. Among the 45 animals of Group I in Experiment I, two spawned another four times within 181 days after their first spawn, with a 45-day average interval between spawnings. The other two individuals spawned only one other time, and the mean interval for them is 181 days (Table 3). This variation is further reflected by the result from Experiment II in which animals were not reared in groups but individually. Among examined two hermaphrodites, 16 males and 19 females, we observed that 8 lancelets (Male #5, 6 and 9, and Female #2, 4, 11, 14 and 19) could spawn again within 35 days. But some animals, such as Hermaphrodite #10, Male #11, 13, and 18 and Female #3, 6 and 7, needed more than 100 days to spawn again (Table 3). By continuously collecting samples in the field, Zhang *et. al* (2007) observed that some adult *B. belcheri* could spawn twice within one breeding season, and they interpreted this pattern as repeated spawnings by 2 or 3-year old lancelet individuals. We don't know whether the variation in spawning intervals in our study was caused by the age or genetic reasons. There was no obvious difference in the body lengths of individuals with short intervals compared to those with long intervals (data not shown). Considering the great variations in the DNA and growth rate among individuals, we would like to suggest that different genetic background is the cause of the difference in reproductive cycles. If true, it would be of great significance to breed the strains with shorter spawning intervals and higher fertility.

### Implications of our studies on *B. belcheri* to other lancelet species

To date, four species from genus *Branchiostoma* are commonly used for studies of developmental biology. Among them, *B. belcheri* and *B. floridae* are sub-tropical species, but *B. japonicum* and *B. lanceolatum* belong to temperate ones [27]. Therefore it is reasonable that *B. belcheri* shows more similarities with *B. floridae* with respect to spawning behavior, and the same is true for *B. lanceolatum* and *B. japonicum*. For example, both *B. belcheri* and *B. floridae* need a relatively higher temperature (above 24°C) to develop and ripen their gonads than that required by *B. japonicum* and *B. lanceolatum* (approximately 15–21°C), and the former two species have longer breeding seasons (July to September for *B. belcheri* in Xiamen [15] and the beginning of May to the beginning of September for *B. floridae* in Florida [27]) than the later two species (the end of May to the end of July for *B. lanceolatum* in Argeles-sur-Mer [22] and June and July for *B. japonicum* in Japan [26]). There are also several similarities which are common among all these four species. First, in natural populations, more than one spawning by all or some individuals within a given breeding season has been suggested, with *B. floridae* having the shortest interval between consecutive spawning of about 1–2 weeks [15], [21], [26], [27]. Second, for the same species, the breeding season varies considerably depending on the water temperature where they live. Usually, a higher temperature corresponds to an earlier and longer breeding season [27]. Considering these observations, we primarily believe that *B. floridae*, *B. japonicum* and *B. lanceolatum* could also spawn repeatedly all-year round if they were cultured in appropriate environmental conditions. However our studies on *B. japonicum* detected a more complex reproductive pattern. Only 50.6% (44/87), 18.8% (16/85) individuals could spawn twice from 14^th^ November 2011 to 16^th^ May 2012 when *B. japonicum* were cultured in groups at 25–27°C, whereas none (0/78) of *B. japonicum* individuals spawned repeatedly when they were cultured at room temperature (18–22°C) or 18–19°C. Interestingly, the initial spawning date of the animals was advanced about 1–2 months in those cultivated at the high temperature (25–27°C) than those cultured under lower temperatures (18–22°C) or from the field. This result suggest that more investigations on potential repeated spawning are required for those lancelets living in temperate seas such as *B. japonicum* and *B. lanceolatum*.

## Concluding Remarks

The phylogenetic position of amphioxus, together with its relatively simple and evolutionarily conserved embryogenesis, morphology and genome content, led to its use as a model for studies of vertebrate evolution and for understanding the elaboration of entire chordate lineage. Although several important advances have been made recently by the researchers, there are still several limitations for amphioxus to be used as a laboratorial model system. Among them, the rare supply of living embryonic materials, which currently are available only in the numbered days of the breeding season, is a major drawback. This limits not only *in vivo* studies on amphioxus embryos, but also the introduction of experimental techniques into amphioxus researches. In this study, we have developed a method for keeping Chinese amphioxus *B. belcheri* spawning consecutively in capacity, which now makes the living embryonic materials of *B. belcheri* available year around. In the method, we emphasized that a relatively high water temperature (25–28°C), and sufficient food supply, as well as low stocking density and cleanness of settlement substratum, are key factors for consecutive breeding. The results also have implications for the cultivation of other amphioxus species.
